# Comparison of Optical Biometric Parameters Between Phakic and Pseudophakic Eyes: A Retrospective Clinical Study

**DOI:** 10.3390/medicina61122155

**Published:** 2025-12-03

**Authors:** Merve Subaşı, Veysi Yıldız, Muhammed Batur

**Affiliations:** 1 Department of Ophthalmology, Bayburt State Hospital, 69000 Bayburt, Turkey; 2 Department of Ophthalmology, Medical Faculty, Yuzuncu Yıl University, 65090 Van, Turkey; 3 Urartu Eye Center, 65080 Van, Turkey

**Keywords:** pseudophakia, anterior eye segment, optical biometry, postoperative refractive error

## Abstract

*Background and Objective:* To evaluate the effect of pseudophakia on anterior chamber depth and other ocular biometric parameters, and to analyze its relationship with age, sex, and axial length. *Materials and Methods:* Optical biometry data from 2372 eyes of 1186 patients—each with one pseudophakic and one phakic eye—were retrospectively analyzed using the Lenstar LS 900^®^. Recorded parameters included axial length (AL), central corneal thickness (CCT), aqueous depth (AD), anterior chamber depth (ACD), lens thickness (LT), keratometry values, and white-to-white distance (WTW). Interocular differences and correlations among variables were statistically assessed. *Results:* The mean age was 62.8 ± 16.0 years (range, 11–92), and 57.1% were male. Compared with phakic eyes, pseudophakic eyes showed significantly lower AL, WTW, and K1 values (*p* < 0.01, *p* < 0.001) and higher CCT, AD, ACD, and astigmatism (AST) values (*p* < 0.001). No significant difference was observed in K2 (*p* > 0.05). In both phakic and pseudophakic eyes, males had higher AL, AD, WTW, and ACD and lower K1 and K2 values than females (*p* < 0.001, *p* < 0.05). Increasing age was associated with decreased AL and CCT. In phakic eyes, ACD and AD were negatively correlated with age, whereas in pseudophakic eyes, the correlation was positive. *Conclusions:* Cataract surgery and intraocular lens implantation significantly alter anterior segment structures. Pseudophakia mainly affects ACD and AD, which may be related to postoperative intraocular pressure reduction and may improve the precision of IOL power calculations.

## 1. Introduction

Cataract is simply defined as the opacification of the crystalline lens. Genetic and environmental factors play a role in its formation. Frequently, high–molecular-weight proteins accumulate, and changes occur in the microarchitecture of the lens [1,2]. Cataract surgery is one of the most commonly performed procedures worldwide. The most ideal method is intraocular lens (IOL) implantation immediately after the removal of the cataractous lens [3]. Both the development of cataract in the crystalline lens and the implantation of an IOL may lead to certain changes in the anterior segment. Several methods, such as anterior segment optical coherence tomography, ultrasound biometry, and optical biometry, can be used to evaluate these changes in ocular biometric parameters. In our study, we preferred the optical biometry method.

The Lenstar LS 900^®^ (Haag-Streit AG, Bern, Switzerland) used in this study is a noninvasive biometry device with optical low-coherence reflectometry capability. It measures ocular distances by utilizing the effect of time-domain interferometry, or the coherent superposition of light waves. Reflections from different intraocular structures, such as the lens, cornea, and retina, are superimposed interferometrically onto the reflections from the reference arm. When the measurement beam is fixated by the patient and perpendicular to the interface, an interference signal is generated from a reflective interface. The device can perform 16 consecutive scans per measurement without the need for realignment. It enables the calculation of ocular biometric parameters such as axial length (AL), central corneal thickness (CCT), aqueous depth (AD), anterior chamber depth (ACD), lens thickness (LT), keratometry values, and white-to-white (WTW) distance [4,5]. Since optical biometry is a non-contact method, it minimizes the rate of human-induced error. Due to its higher repeatability and lower rates of postoperative refractive error, it has increasingly become the gold standard, particularly for IOL power calculation [6].

The effects of cataract surgery on the anterior segment have previously been evaluated in different patient groups using various methods [7,8,9,10,11]. In our study, we evaluated patients by optical biometry, in which one eye had undergone cataract surgery with IOL implantation (pseudophakic), while the fellow eye had not undergone surgery (phakic). The aim of the study was to investigate the effect of pseudophakia on anterior chamber depth and other ocular biometric measurements, as well as its association with age, sex, and axial length. Unlike previous studies, the inclusion of a larger number of participants allows for a clearer demonstration of the impact of cataract surgery on the anterior segment.

## 2. Materials and Methods

Our study was conducted at the Department of Ophthalmology, Dursun Odabaş Medical Center, Van Yüzüncü Yıl University, by retrospectively reviewing the data of patients who underwent optical biometry between 2016 and 2024. Prior to the initiation of the study, approval was obtained from the local ethics committee (Date: 26 August 2025, Decision No: 2025/08-21), and the research was carried out in accordance with the principles of the Declaration of Helsinki.

Between 2016 and 2024, the records of approximately 22,700 patients measured with the Lenstar LS 900^®^ (Haag-Streit AG, Bern, Switzerland) optical biometry device were retrospectively reviewed. A total of 1186 patients, each with one eye that had undergone cataract surgery with IOL implantation (pseudophakic) and the fellow eye remaining phakic, were included in the study, yielding 2372 eyes (1186 phakic and 1186 pseudophakic eyes). Biometric measurements were obtained from the most recent clinical visit, during which both the phakic and pseudophakic eyes were measured simultaneously. Because the study was retrospective, the exact dates of the cataract surgeries were not available in the medical records. The age range was set between 10 and 100 years. Patients who remained aphakic following cataract surgery and those in whom complete biometric measurements could not be obtained by the device were excluded from the study.

All pseudophakic eyes underwent standard phacoemulsification with implantation of a one-piece hydrophobic acrylic monofocal IOL (AcrySof SA60AT, Alcon Laboratories, Fort Worth, TX, USA) through a 2.2 mm clear corneal incision located at the 12 o’clock position. In cases where posterior capsular rupture occurred during surgery, a three-piece hydrophobic acrylic IOL (AcrySof MA60AC, Alcon Laboratories, Fort Worth, TX, USA) was implanted in the ciliary sulcus, and the main incision was enlarged as needed to allow safe lens implantation. Because this study was retrospective, the exact number of eyes in which a three-piece IOL was used could not be determined due to incomplete surgical records.

For the phakic and pseudophakic eyes of the included patients, the parameters measured by optical biometry—axial length (AL), central corneal thickness (CCT), aqueous depth (AD), anterior chamber depth (ACD), white-to-white distance (WTW), the flattest keratometry (K1), the steepest keratometry (K2), and astigmatism values—were recorded. The differences between the two groups were statistically analyzed.

### Statistical Analysis

For the evaluation of the findings obtained in the study, statistical analyses were performed using SPSS version 27 (Statistical Package for the Social Sciences; IBM Corp., Armonk, NY, USA). The normality of the distribution of scores obtained from each continuous variable was examined using descriptive methods (skewness–kurtosis and standard deviation/mean ratio), graphical methods (Q–Q plots and histograms), and statistical testing (Kolmogorov–Smirnov test). Categorical variables were presented as frequencies (n, %), while continuous variables were presented as mean and standard deviation. Comparisons between two independent groups for continuous variables were performed using the Independent Samples *t*-test. Comparisons between two dependent groups for continuous variables were conducted using the Paired Samples *t*-test. The relationships between two continuous variables were analyzed using the Pearson correlation test. Receiver operating characteristic (ROC) analysis was used to assess the diagnostic ability of optical biometry parameters. For the diagnostic performance of the parameters in identifying the operated eye, the area under the ROC curve (AUC), sensitivity, specificity, and accuracy values were calculated. The optimal cutoff level for the parameters was determined using the Youden index. Results were evaluated within a 95% confidence interval, and statistical significance was defined as *p* < 0.05.

## 3. Results

A total of 1186 patients who had undergone unilateral cataract surgery were included in the study. The mean age of the participants was 62.81 ± 15.99 years (range: 11–92 years), and 57.1% were male. The optical biometry measurement results of the phakic and pseudophakic eyes are presented in Table 1. Compared with phakic eyes, pseudophakic eyes had significantly lower AL, WTW, and K1 optical biometric values (*p* < 0.01 and *p* < 0.001), whereas CCT, AD, ACD, and astigmatism (AST) measurements were significantly higher (*p* < 0.001). However, no statistically significant difference was found in K2 values (*p* > 0.05).

When the optical biometry results of phakic and pseudophakic eyes were analyzed according to sex (Table 2), the statistical findings between phakic and pseudophakic eyes were found to be similar in both female and male patients (*p* < 0.001; *p* < 0.01; and *p* < 0.05). However, in within-group comparisons by sex, males showed significantly higher AL, AD, WTW, and ACD values in both phakic and pseudophakic eyes, whereas K1 and K2 values were significantly lower (*p* < 0.001 and *p* < 0.05).

When the correlation between optical biometry parameters and age was examined (Table 3), an age-related decrease in AL was observed in both eyes (phakic eye: r = −0.071, *p* = 0.015; pseudophakic eye: r = −0.126, *p* < 0.001). Similarly, CCT was negatively correlated with age (phakic eye: r = −0.179, *p* < 0.001; pseudophakic eye: r = −0.173, *p* < 0.001). In phakic eyes, ACD and AD showed a significant negative correlation with age (ACD: r = −0.325, *p* < 0.001; AD: r = −0.310, *p* < 0.001), whereas in pseudophakic eyes, these parameters were positively correlated (ACD: r = 0.216, *p* < 0.001; AD: r = 0.225, *p* < 0.001).

According to the results of the ROC curve analysis, the optical biometry parameters that most accurately distinguished pseudophakic eyes from phakic eyes were AD (AUC = 0.935, cutoff point = 3.41) and ACD (AUC = 0.936, cutoff point = 3.92). For the cutoff point of aqueous depth, the sensitivity, specificity, and accuracy were calculated as 84.2%, 96.1%, and 90.1%, respectively, while for ACD, these values were 84.7%, 95.7%, and 90.2%, respectively.

The correlation between optical biometry parameters is presented in detail in Table 4.

## 4. Discussion

In cataract surgery, the thick crystalline lens is removed and replaced with a thinner artificial intraocular lens. This alteration, along with factors such as corneal incisions performed during surgery, leads to significant changes in anterior segment parameters. The aim of our study was to evaluate the effect of pseudophakia on optical biometry parameters, particularly anterior chamber depth and aqueous depth. In 1186 patients and 2372 eyes, with one eye phakic and the fellow eye pseudophakic, we observed significant changes in anterior segment parameters.

Anterior chamber depth (ACD) is defined as the distance between the corneal epithelium and the anterior surface of the crystalline lens, whereas aqueous depth (AD) is obtained by measuring this distance with reference to the corneal endothelium [12]. Both ACD and AD measurements can be clinically useful in many aspects, such as IOL power calculation or screening patients with narrow-angle glaucoma. In our study, we found that ACD and AD values were significantly higher in pseudophakic eyes compared with phakic eyes (4.69 ± 0.79 mm vs. 3.20 ± 0.43 mm; 4.17 ± 0.79 mm vs. 2.68 ± 0.43 mm, respectively). This finding was consistent with several previous studies that demonstrated an increase in ACD following cataract surgery [8,11,13,14,15].

The ROC curve provides a graphical representation of the relationship between sensitivity and specificity and allows comparison of the diagnostic performance of different parameters. In the present study, ROC analysis was included to determine which optical biometry variables most accurately differentiate pseudophakic from phakic eyes. This has important clinical implications because, in real-world practice, pre-operative biometry may be unavailable, incomplete, or unreliable, particularly in patients referred from other centers or those with complex medical histories. Identifying the parameters with the highest discriminative power can therefore assist clinicians in estimating surgical status and understanding postoperative anatomical changes when pre-operative measurements are missing. In our cohort, ACD and AD demonstrated excellent diagnostic performance, with AUC values of 0.936 and 0.935, respectively, confirming that these parameters undergo the most prominent postoperative modifications following cataract surgery.

The increase in ACD observed in pseudophakic eyes has been explained by several mechanisms. One explanation is that, following removal of the crystalline lens, the iris undergoes an approximate 10-degree posterior angular rotation, with the elimination of relative pupillary block, particularly in cases with narrow angles [11]. Another mechanism is the posterior shift of the IOL over time due to fibrosis and opacification of the posterior capsule. This may influence the effective lens position (ELP), which is highly important in postoperative refractive error. It has been suggested that ACD can also serve as an indicator for ELP [16,17]. The increase in ACD may be associated with postoperative hyperopic shift; therefore, surgeons may aim for a postoperative myopic refraction [14]. Similarly, a study conducted in patients with pseudoexfoliation (PEX) syndrome demonstrated that ACD increased postoperatively, and within the first six months, the IOL shifted posteriorly, resulting in a slight hyperopic shift [18]. In another study, a greater increase in ACD was observed after cataract surgery, particularly in patients with initially shallow anterior chambers and shorter axial lengths, and this marked change in ACD was associated with a myopic shift. It was emphasized that ACD could serve as a predictor of refractive error [15,19]. At the same time, ACD is incorporated into many formulas used for IOL power calculation (such as Haigis, Barrett Universal II, and Olsen). Therefore, accurate measurement of ACD is of critical importance for postoperative refraction.

Previous studies have shown that the Lenstar LS 900^®^ (Haag-Streit AG, Switzerland) is considered reliable in terms of repeatability and compatibility with ultrasonographic devices [20,21]. This has been demonstrated particularly in phakic eyes; however, in pseudophakic eyes, it has been emphasized that, as with other newly introduced devices (e.g., Pentacam, IOLMaster 700), certain features still require improvement. Although the type of IOL used has less influence on the measurements in pseudophakic eyes, the formula applied for the calculation is of greater importance [22].

Since our study was not designed prospectively, we were unable to directly observe preoperative and postoperative changes. However, we compared data obtained from the phakic and pseudophakic eyes of the same patients. The large sample size is an important advantage in this respect. In particular, for patients who underwent cataract surgery in one eye long ago and lack preoperative biometry data, our study may provide guidance for accurate IOL power calculation and prediction of refractive error. However, in patients where optical biometry cannot be obtained due to dense cataract, postoperative measurements in the pseudophakic eye may serve as a guide for preoperative IOL selection in the fellow eye. Therefore, accurate IOL measurements in pseudophakic eyes are of great importance.

In a study conducted with the IOLMaster, refractive error was found to be higher in pseudophakic eyes compared with phakic eyes, and depending on the formula used, a correction of 0.20 to 0.65 D in the hyperopic direction was recommended to achieve the target refraction [22].

The reduction in intraocular pressure (IOP) following cataract surgery is associated with several factors. One of these is the removal of the thick crystalline lens and its replacement with a thinner IOL. In addition, the increase in ACD and AD facilitates easier access of the aqueous humor to the trabecular meshwork. Moreover, changes in iris position reduce resistance within the trabecular meshwork. Previous studies have demonstrated that the increase in angle parameters accompanying the postoperative rise in ACD is associated with a decrease in IOP [7,9,10]. Further studies are needed to determine the predictive role of ACD on IOP in pseudophakic eyes after cataract surgery.

In our study, axial length was found to be significantly shorter in pseudophakic eyes compared with phakic eyes (23.32 ± 1.45 mm vs. 23.48 ± 1.31 mm, respectively). This finding contradicts some studies that have reported no effect of cataract surgery on AL [14,23]. One study suggested that the observed decrease in AL may not be real and could be related to several factors. The first is the change in astigmatism and mean keratometry (Km) associated with the corneal incision. In our study, while K1 was significantly lower in pseudophakic eyes (*p* < 0.001), astigmatism was significantly higher (1.30 ± 1.14 D vs. 1.09 ± 1.04 D). We may attribute the AL change observed in our study to these alterations in corneal curvature.

A second factor is the reduction in total ocular volume following removal of the crystalline lens, which may contribute to a decrease in AL. Another possibility is that no true change exists, and the shorter values observed postoperatively result from inaccurate estimations during measurement. Since AL is a key component of all IOL power calculation formulas, such measurement errors can increase the rate of refractive error. To prevent this, it has been emphasized that different sound velocities in various tissues should be taken into account during ultrasonic measurements [24], and subsequently, different correction factors have been proposed for different lens materials [25]. For example, the IOLMaster automatically adds 0.1 mm in pseudophakic eyes as a correction. Finally, the shorter AL observed in pseudophakic eyes may be related to inaccuracies in preoperative measurements, particularly in eyes with dense cataracts [23]. These factors may explain the shorter AL measurements observed in pseudophakic eyes in our study. Correction factors previously described may be insufficient for accurate AL measurement today, and further studies are needed.

In the correlation analysis between AL and ACD/AD, a stronger positive correlation was observed particularly in phakic eyes. As axial length increased, both ACD and AD also increased. In a systematic review and meta-analysis examining the correlation of ocular biometric parameters, a moderately varying but generally positive correlation between AL and ACD was emphasized [26]. This analysis is supported by numerous studies [27,28,29,30]. In addition, two of these studies, similar to our findings, also reported a positive correlation between ACD and WTW [29,30].

In our study, CCT was found to be higher in pseudophakic patients compared with phakic patients (522.46 ± 45.88 μm vs. 517.82 ± 38.37 μm, respectively). Unlike our results, although a short-term increase in CCT after surgery has been reported due to endothelial damage, no significant change was observed in long-term outcomes [10,11,31,32]. In one study, however, a decrease was observed at the end of three years compared with baseline values [14]. In our study, no data were available regarding the timing of cataract surgery. Furthermore, while other studies compared preoperative and postoperative values, our study compared the phakic and pseudophakic fellow eyes of the same patients. Considering that some of our patients underwent cataract surgery due to trauma, the higher CCT measurements in pseudophakic eyes may be related to endothelial damage. In our study, no clinically or statistically significant correlation was observed between CCT and AL. This finding is supported by the study of Popov et al. [33], whereas another study reported a significant negative correlation in myopic eyes, showing that CCT decreased as AL increased [34]. Overall, CCT has been found to have limited association with other optical biometry parameters, and although statistically significant correlations with AD and ACD were observed, they were clinically weak.

Astigmatism is determined by the difference between K1 and K2. As noted above, in our study, astigmatism values were found to be higher in pseudophakic patients. However, a recent study comparing patients before and after cataract surgery reported no significant changes in anterior corneal astigmatism, posterior corneal astigmatism, or total astigmatism three months postoperatively [35]. In the correlation analysis conducted in the later stages of the study, an increasing trend in postoperative astigmatism was observed with greater AL, particularly in older individuals. This finding has been explained in the literature by the corneal biomechanical weakness, reduced corneal thickness, and greater susceptibility to deformation that accompany increased AL [36,37].

Similarly, in our study, we found a positive correlation between axial length and astigmatism, which was more pronounced in the pseudophakic group. In another study with a three-year follow-up, a significant change in K1 was observed only during the first year, whereas no significant change in K2 was noted throughout the follow-up period [14]. The higher level of astigmatism observed in pseudophakic patients in our study may be attributable to several factors, including the type of blade used, the location of the corneal incision, and the time elapsed after surgery.

In our study, the statistical findings between phakic and pseudophakic eyes were similar in both male and female patients. In addition, AL, AD, WTW, and ACD were found to be significantly higher in males, whereas K1 and K2 values were significantly higher in females. This is consistent with a study presenting preoperative optical biometry data in a large sample, in which AL and ACD were significantly higher in males, while the cornea was found to be steeper in females [6]. In particular, the higher AL and ACD values observed in males have also been demonstrated in previous studies [30,33,38,39,40].

When we examined the correlation of age with optical biometry parameters, we found a significant decrease in AL and CCT with increasing age in both phakic and pseudophakic patients. The reduction in CCT has been attributed to age-related thinning of the epithelium, stroma, and endothelium. Several studies have demonstrated this negative correlation [41,42,43]. The decrease in axial length with advancing age has also been demonstrated in previous studies, supporting our findings [40,44,45].

However, several factors, such as patients’ educational level, body height, and device measurement errors related to age-associated lenticular changes, may contribute to variations in axial length. It has been emphasized that this may lead to erroneous reductions in AL, particularly in cross-sectional studies [46]. In other words, the observed decrease may not reflect a true change, and further longitudinal studies are needed.

In our study, we found that ACD and AD decreased with age in phakic patients, whereas a positive correlation with age was observed in pseudophakic patients. Similarly to other studies, we attribute this finding in phakic patients to the age-related increase in lens thickness, which reduces ACD [47,48].

The novelty of our study lies in the large sample size and the direct comparison of fellow phakic and pseudophakic eyes in the same patients, which eliminates many inter-individual anatomical differences that affect anterior segment measurements. In addition, we demonstrated that ACD and AD have excellent diagnostic ability in distinguishing pseudophakic from phakic eyes, with AUC values above 0.93. To our knowledge, no previous study with a similarly large cohort has quantified this discriminative performance using ROC analysis. These findings highlight the clinical relevance of anterior chamber parameters not only as postoperative anatomical indicators but also as reliable markers that may assist clinicians when pre-operative measurements are missing or unreliable.

The main limitation of our study is its retrospective design and the lack of access to preoperative data of the patients. In addition, the measurements obtained in pseudophakic eyes may vary depending on the time elapsed after surgery. Our study did not include data on when the patients underwent surgery. Furthermore, the fact that the surgeries were not performed by a single surgeon and that a single type of IOL was not used represents another important limitation.

## 5. Conclusions

Cataract surgery leads to significant and clinically relevant alterations in anterior segment biometry. Among all parameters, anterior chamber depth and aqueous depth showed the largest postoperative changes and the highest discriminative performance (AUC > 0.93) in distinguishing pseudophakic from phakic eyes. Given the large sample size and the comparison of fellow eyes, our findings provide robust evidence that ACD and AD can be used as reliable anatomical indicators in both postoperative assessment and in cases where pre-operative biometry is unavailable.

## Figures and Tables

**Table 1:** Comparison of Optical Biometry Measurements Between Phakic and Pseudophakic Eyes.

Measurements	n	Phakic Eyes Mean ± SD	Pseudophakic Eyes Mean ± SD	Mean Difference (95% CI)	*p*-Value ^a^
AL (mm)	1173	23.48 ± 1.31	23.32 ± 1.45	−0.16 [−0.21; −0.11]	<0.001 *
CCT (µm)	1186	517.82 ± 38.37	522.46 ± 45.88	4.64 [2.88; 6.41]	<0.001 *
AD (mm)	1186	2.68 ± 0.43	4.17 ± 0.79	1.49 [1.44; 1.54]	<0.001 *
WTW (mm)	1156	11.81 ± 0.60	11.76 ± 0.62	−0.05 [−0.08; −0.02]	0.004 **
ACD (mm)	1186	3.20 ± 0.43	4.69 ± 0.79	1.50 [1.45; 1.54]	<0.001 *
K1 (D)	1128	43.35 ± 1.80	43.08 ± 2.00	−0.27 [−0.35; −0.19]	<0.001 *
K2 (D)	1128	44.44 ± 1.97	44.38 ± 2.18	−0.06 [−0.15; 0.04]	0.230
AST (D)	1128	1.09 ± 1.04	1.30 ± 1.14	0.21 [0.13; 0.29]	<0.001 *

* *p* < 0.001; ** *p* < 0.01; ^a^, Paired samples *t* test; SD, Standard Deviation; CI, Confidence Interval.

**Table 2:** Comparison of Optical Biometry Measurements Between Phakic and Pseudophakic Eyes According to Sex.

			Phakic Eyes	Pseudophakic Eyes	Difference	
Measurements	Gender	n	Mean ± SD	Mean ± SD	Mean [95% CI]	*p*-Value ^a^
AL (mm)	Female	503	23.31 ± 1.60	23.08 ± 1.64	−0.23 [−0.31; −0.15]	<0.001 *
	Male	670	23.60 ± 1.03	23.50 ± 1.26	−0.11 [−0.17; −0.05]	0.001 **
	*p*-value ^b^		<0.001 *	<0.001 *		
CCT (μm)	Female	509	517.64 ± 37.27	520.98 ± 43.38	3.34 [0.69; 6.00]	0.014 ***
	Male	677	517.95 ± 39.20	523.57 ± 47.68	5.62 [3.26; 7.98]	<0.001 *
	*p*-value ^b^		0.888	0.335		
AD (mm)	Female	509	2.58 ± 0.42	4.11 ± 0.80	1.53 [1.46; 1.60]	<0.001 *
	Male	677	2.75 ± 0.42	4.22 ± 0.78	1.47 [1.40; 1.53]	<0.001 *
	*p*-value ^b^		<0.001 *	0.018 ***		
WTW (mm)	Female	495	11.73 ± 0.55	11.67 ± 0.61	−0.05 [−0.10; 0.00]	0.041 ***
	Male	661	11.87 ± 0.62	11.83 ± 0.63	−0.05 [−0.09; 0.00]	0.039 ***
	*p*-value ^b^		<0.001 *	<0.001 *		
ACD (mm)	Female	509	3.10 ± 0.42	4.63 ± 0.80	1.53 [1.46; 1.60]	<0.001 *
	Male	677	3.27 ± 0.42	4.74 ± 0.78	1.47 [1.41; 1.53]	<0.001 *
	*p*-value ^b^		<0.001 *	0.015 ***		
K1 (D)	Female	479	43.89 ± 1.85	43.63 ± 2.13	−0.27 [−0.40; −0.13]	<0.001 *
	Male	649	42.95 ± 1.65	42.68 ± 1.80	−0.27 [−0.37; −0.17]	<0.001 *
	*p*-value ^b^		<0.001 *	<0.001 *		
K2 (D)	Female	479	44.96 ± 2.08	44.87 ± 2.39	−0.09 [−0.26; 0.07]	0.270
	Male	649	44.05 ± 1.79	44.02 ± 1.94	−0.03 [−0.14; 0.08]	0.571
	*p*-value ^b^		<0.001 *	<0.001 *		
AST (D)	Female	479	1.07 ± 1.07	1.24 ± 1.06	0.17 [0.06; 0.28]	0.002 **
	Male	649	1.10 ± 1.03	1.34 ± 1.20	0.24 [0.13; 0.35]	<0.001 *
	*p*-value ^b^		0.598	0.149		

* *p* < 0.001; ** *p* < 0.01; *** *p* < 0.05; ^a^, Paired samples *t* test; ^b^, Independent samples *t*-test; SD, Standard Deviation; CI, Confidence Interval.

**Table 3:** Correlation Between Optical Biometry Measurements and Age.

	Age			
	Phakic Eyes		Pseudophakic Eyes	
Measurements	r	*p*	r	*p*
AL	−0.071	0.015 **	−0.126	<0.001 *
CCT	−0.179	<0.001 *	−0.173	<0.001 *
AD	−0.310	<0.001 *	0.225	<0.001 *
WTW	−0.219	<0.001 *	−0.208	<0.001 *
ACD	−0.325	<0.001 *	0.216	<0.001 *
K1	0.069	0.018 **	0.043	0.148
K2	0.041	0.158	−0.025	0.391
AST	−0.041	0.160	−0.124	<0.001 *

* *p* < 0.01; ** *p* < 0.05; r, Pearson correlation test.

**Table 4:** Correlation Between Continuous Variables.

	**Phakic Eyes**						
	**AL**	**CCT**	**AD**	**WTW**	**ACD**	**K1**	**K2**
AL	N/A						
CCT	−0.034						
AD	0.316 *	−0.025					
WTW	0.234 *	0.033	0.340 *				
ACD	0.312 *	0.065 **	0.996 *	0.343 *			
K1	−0.411 *	−0.096 *	−0.092 *	−0.317 *	−0.100 *		
K2	−0.331 *	−0.090 *	−0.056	−0.339 *	−0.063 **	0.850 *	
AST	0.085 *	−0.004	0.054	−0.091 *	0.053	−0.125 *	0.416 *
	**Pseudophakic Eyes**						
	**AL**	**CCT**	**AD**	**WTW**	**ACD**	**K1**	**K2**
AL	N/A						
CCT	−0.046						
AD	0.087 *	−0.117 *					
WTW	0.151 *	0.014	0.088 *				
ACD	0.084 *	−0.060 **	0.998 *	0.090 *			
K1	−0.287 *	−0.146 *	0.120 *	−0.253 *	0.112 *		
K2	−0.229 *	−0.132 *	0.053	−0.264 *	0.045	0.855 *	
AST	0.065 **	0.004	−0.110 *	−0.061 **	−0.111 *	−0.120 *	0.413 *

* *p* < 0.01; ** *p* < 0.05; N/A: Not available.

## Data Availability

The raw data supporting the conclusions of this article will be made available by the authors upon request.
